# Synthesis and Characterization of *N*-Substituted Polyether-*Block*-Amide Copolymers

**DOI:** 10.3390/ma14040773

**Published:** 2021-02-06

**Authors:** Jyun-Yan Ye, Kuo-Fu Peng, Yu-Ning Zhang, Szu-Yuan Huang, Mong Liang

**Affiliations:** 1 Department of Applied Chemistry, National Chia-Yi University, Chia-Yi 600, Taiwan; s1000268@gm.pu.edu.tw (J.-Y.Y.); s1080246@mail.ncyu.edu.tw (Y.-N.Z.); 2 Department of Footwear Technology, Footwear & Recreation Technology Research Institute, Taichung 407, Taiwan; 0828@bestmotion.com (K.-F.P.); 0150@bestmotion.com (S.-Y.H.)

**Keywords:** polyether-*block*-amide, *N*-substituted nylon elastomers, polyamide 6, crystallinity, thermal properties

## Abstract

A series of *N-*substituted polyether-*block*-amide (PEBA-X%) copolymers were prepared by melt polycondensation of nylon-6 prepolymer and polytetramethylene ether glycol at an elevated temperature using titanium isopropoxide as a catalyst. The structure, thermal properties, and crystallinity of PEBA-X% were investigated using nuclear magnetic resonance spectroscopy, Fourier-transform infrared spectroscopy, differential scanning calorimetry, wide angle X-ray diffraction, and thermogravimetric analysis. In general, the crystallinity, melting point, and thermal degradation temperature of PEBA-X% decreased as the incorporation of *N*-methyl functionalized groups increased, owing to the disruption caused to the structural regularity of the copolymer. However, in *N*-acetyl functionalized analogues, the crystallinity first dropped and then increased because of a new γ form arrangement that developed in the microstructure. After the cross-linking reaction of the *N*-methyl-substituted derivative, which has electron-donating characteristics, with poly(4,4′-methylenebis(phenyl isocyanate), the decomposition temperature of the resulting polymer significantly increased, whereas no such improvements could be observed in the case of the electro-withdrawing *N*-acetyl-substituted derivative, because of the incompleteness of its cross-linking reaction.

## 1. Introduction

Polyether-*block*-amide (PEBA) is a block copolymer elastomer comprising rigid polyamide blocks and flexible polyether blocks [1,2]. By controlling the mole ratio of the polyamide blocks to that of polyether blocks and without adding any plasticiser, we can obtain elastomers with different degrees of hardness and elasticity, i.e., highly tough to ultra-soft, and very strong to ultra-pliable [3]. These unique elastomers combine perfectly the stiffness of polyamide and the pliability of polyether; they are soft and have good low-temperature flexibility, and are mainly used in soles, medical tubes, aerospace parts, and in the chemical separation [4,5,6,7].

PEBA has been produced using solvent polymerisation, interfacial polymerisation, bulk polymerisation, etc. [8,9,10,11,12]. Zdrahala et al. [8] used solvent polymerisation to obtain poly(*m*-phenylene isophthalamide-*b*-ethylene oxide) by using *N,N*-dimethylacetamide as the solvent, and *m*-phenylenediamine and isophthaloyl chloride as monomers for the polyamide segment and poly(ethylene oxide) of different molecular weights. Gaymans et al. [9] prepared nylon 4,6-polytetramethylene oxide segmented block copolymers using *N*-methyl-2-pyrrolidone at a pressure of 5 bar and a temperature of 200–210 °C. The prepolymers in their solid state were then postcondensed at 255 °C to obtain PEBAs of high molecular weight. Castaldo et al. [10] reacted low-molecular-weight poly(oxyalkylene glycol) with a large excess of dicarboxylic acid chloride to obtain acid chloride end-capped polyether which was thereafter subjected to interfacial polycondensation with diamines to obtain PEBA copolymers in 50–60% yields. Kimura et al. [11] conducted bulk polymerisation of 1:1 nylon salts of polyethylene oxide-acids (PEO-acids) with diamines at 270 °C in a vacuum environment to prepare PEBA copolymers. On the other hand, Rasmussen and Smith [12] used piperasine, sebacic acid, and commercialised Jeffamine^@^ D-2000 to obtain PEBA in a partial vacuum environment in which nitrogen flowed at a temperature between 220 °C and 245 °C. Most of the raw materials used in PEBA are based mainly on long-chain aliphatic polyamides, such as nylon-12 and nylon-10,10. Few studies have been conducted on PEBA that had nylon-6 as the raw material, mainly because the crystallinity of nylon-6 is higher than that of any other polymer. If the crystallinity of nylon-6 can be reduced, the materials could have a lower processing temperature and higher resistance to abrasion, thereby bridging the gap between traditional polyamides and polyethylenes. Xu et al. [13] used the monomer casting method to prepare various nylon-6/PEO blends using in situ polymerisation and found that the addition of PEO decreased the crystallisation ability of the nylon-6 matrix although it improved the crystallisation ability of the PEO phase. Rwei et al. [14] synthesized various types of low melting temperature polyamide 6 copolyamides with aliphatic/aromatic unitsby using bio-based aromatic *N*^1^,*N*^4^-bis(4-aminobutyl) terephthalamide diamine (BABT) and sebacic salt. Peyravi et al. [15] attempted to tune the block lengths of the hard and soft segments of PEBA by varying the polyamide to polyether weight ratio. X-ray diffractometry showed that two distinct crystalline phases of polyethylene (PE) and polyamide (PA) had formed in the synthesized copolymers and that the increase in PA segments greatly improved the formation of polyamide lamellae crystals.

A series of polyether-*block*-amide copolymers containing varying proportions of *N*-substituted polyamide (PEBA-X%) were synthesized in this study to explore how the structural modification of the polymer will influence the thermal properties and material properties, such as crystallinity, of the newly produced nylon copolymers. Considering the availability of the raw materials for mass production, we choose the already commercialised methyl-caprolactam and acetyl-caprolactam monomers to perform the PEBA synthesis with PA as the hard segment and PE as the soft segment.

## 2. Materials and Methods

### 2.1. Materials

All reagents and solvents were reagent-grade purchasing from commercial companies and used as received. ε-Caprolactam (>99%) and titanium isopropoxide (98%) were obtained from Acros. *N*-methyl-ε-caprolactam (96%) and *N*-acetyl-ε-caprolactam (>99%) were purchased from Tokyo Chemical Industry Co., Ltd. Formic acid (88%) and acetone (98%) were purchased from Fisher Chemical. Adipic acid (99%) was from Showa. Polytetramethylene Ether Glycols were received from Aldrich. Poly(4,4′-methylenebis(phenyl isocyanate),Elasturan^®^ T 6961 (NCO content 23%) was obtained from BASF.

### 2.2. Characterization Techniques

#### 2.2.1. Fourier-Transform Infrared Spectroscopy (FT-IR)

This study used FT-IR (Spectrum Two, Perkin Elmer) to detect experimental samples by pairing them with a reflection element (ZnSe crystal, Model: L160-0115) in a scanning range of 700–4000 cm^−1^.

#### 2.2.2. Nuclear Magnetic Resonance (NMR)

^1^H and ^13^C NMR spectra were recorded on a MercurPlus 300 spectrometer (Varian, Oxford, USA) and a Varian 400 spectrometer (Agilent Technologies, Yarnton, UK), respectively using dimethyl sulfoxide-d_6_ 99.8% as the solvent.

#### 2.2.3. Thermogravimetric Analysis (TGA)

TGA was conducted using a Q50 thermogravimetric analyser (TA Instruments-Waters, New Castle, USA) from TA Instruments. For this analysis, a sample weighing between 5 and 10 mg was placed on a platinum plate in an environment filled with nitrogen (40 mL min^−1^), and the temperature was raised from 50 to 550 °C at a rate of 10 °C min^−1^. The 5% and 10% weight loss temperatures and the main decomposition temperature were determined.

#### 2.2.4. Differential Scanning Calorimetry (DSC)

DSC was conducted using the Q-10 differential scanning calorimeter (TA Instruments-Waters, New Castle, USA) developed by TA Instruments. The instrument was calibrated using a high purity indium standard. All the samples were tested in an environment filled with nitrogen of 99.99% purity. For the standard DSC analysis, samples, which were approximately 3 mg in weight, were placed on hermetically sealed aluminium pans (diameter = 5 mm; TA Instruments). The samples were heated at a rate of 10 °C min^−1^ to 300 °C, cooled to 40 °C, and heated again. The nitrogen gas flow rate was maintained at 50 mL min^−1^.

#### 2.2.5. Powder X-ray Diffraction (XRD)

XRD was conducted using Shimadzu XRD-6000 (Shimadzu, Tokyo, Japan). All the samples were tested at 25 °C using a K α radiation source (Shimadzu, Tokyo, Japan) (λ = 1.54 Å) with Cu as the target metal. The operating voltage was set at 40 KV, electric current at 30 mA, scanning range (2θ) between 10° and 80°, and scanning rate at 3° min^−1^. The analysis was carried out for the scanning range from 10° to 40° and crystallinities of the samples were compared using MDI Jade 6. The degree of crystallinity of each sample was calculated using the following equation:

Crystallinity (%) = [(Total area of the crystalline peaks)/(Total area of all the peaks)] × 100%

### 2.3. Preparation of Nylon Prepolymer

The nylon prepolymer was prepared using ring-opening polymerisation with adipic acid as the catalyst. A 100 mL three neck round bottomed flask, equipped with a magnetic stirrer and a reflux condenser was loaded with different amounts of caprolactam monomer, *N*-methyl-ε-caprolactam, and *N*-acetyl-ε-caprolactam (Table 1). The flask was deoxygenated by degassing and back-flushed with nitrogen three times. It was thereafter slowly heated to 170 °C while being stirred. Next, 5 wt% deionised water and 14 wt% adipic acid were added, and the resulting mixture was heated at 260 °C for 6 h. After the prepolymer had cooled to room temperature, it was stirred with 30 mL of formic acid and to precipitate the product, 500 mL of deionised water was slowly poured into the solution. The precipitated polymer was collected using filtration, washed with deionised water thrice and dried overnight in a vacuum at 70 °C. The installations for preparation of nylon prepolymer and PEBA are shown in Supplementary Materials, Figures S1 and S2.

### 2.4. General Procedure Followed in Preparing PEBA

The polyether-*block*-amide copolymer containing varying proportions of *N*-substituted groups (PEBA-X%) were prepared by melt polycondensation of dicarboxylic acid, *N-s*ubstituted nylon-6 prepolymer, and polytetramethylene ether glycol (PTMEG) in molecular weights of 2000 and 250 at an elevated temperature with titanium isopropoxide as the catalyst. PTMEG was heated to 60 °C in a vacuum for 4 h before being used.

To a stirred mixture of PTMEG 250 (8.25 g, 0.033 mol) and nylon-6 prepolymer (10 g, 0.012 mol) in a nitrogen-filled environment was added 15 mol% of titanium isopropoxide. The resulting reaction mixture was heated to 230 °C for 4 h. After the reactants have cooled to room temperature, 150 mL of reagent-grade acetone was added and stirred evenly for 4 h. The product was filtered, re-washed with acetone, and dried at 80 °C in a vacuum overnight to obtain 10 g of the polymer (PEBA 250). A similar procedure was adopted for all other PEBA-X% polymers but adding with different functionalized ratios of nylon-6 (*N*-acetyl-X%) and nylon-6(*N*-methyl-X%). All nylon-6 prepolymers and PEBA copolymers were isolated as white to pale yellow powders, and the results are summarized in Table 2 and Supplementary Materials, Figure S3.

## 3. Results and Discussion

**Figure 1:**
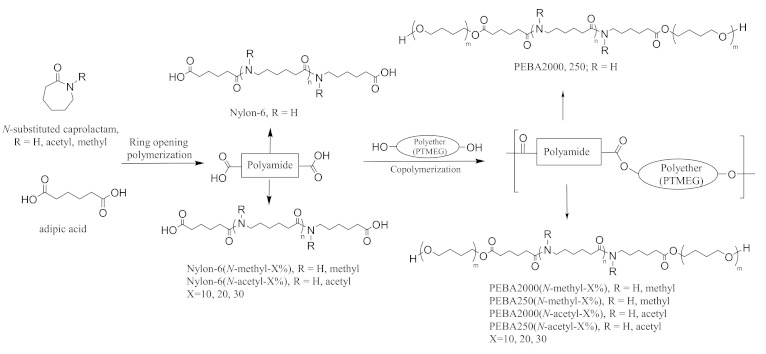
Synthesis route of *N*-substituted polyether-*block*-amide.

Figure 1 shows the approach we used to synthesise *N*-substituted PEBA. First, the caprolactam and different ratios of *N*-methyl-ε-caprolactam or *N*-acetyl-ε-caprolactam were copolymerised with ε-caprolactam to obtain nylon-6 prepolymer (henceforth referred to as nylon-6(*N*-methyl-X%) or nylon-6(*N*-acetyl-X%) using ring-opening polymerisation with adipic acid as the catalyst. The prepolymers were then made to react with PTMEG of molecular weights 2000 and 250 by esterification to form various PEBA copolymers.

### 3.1. Structural Analysis of Nylon Prepolymer

#### 3.1.1. ^1^H NMR

**Figure 2:**
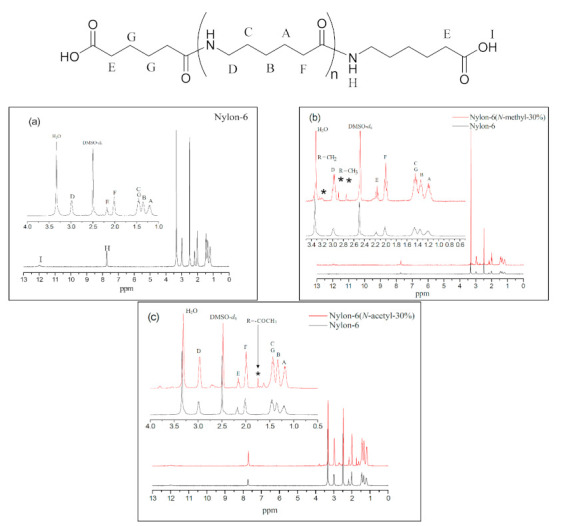
^1^H NMR spectra of (**a**) nylon-6, (**b**) nylon-6 and nylon-6(*N*-methyl-30%), and (**c**) nylon-6 and nylon-6(*N*-acetyl-30%). The asterisk sign represents chemical shift of *N*-substituted group.

All of the products were isolated as a white powder, and their structures were characterised using ^1^H NMR, ^13^C NMR, and FT-IR. According to Figure 2a, which is in respect of nylon-6, the peaks at δ = 1.2 ppm (–***CH_2_***–CH_2_–CONH–, A), δ = 1.3 ppm (–***CH_2_***–CH_2_–CH_2_–CONH–, B), and δ = 1.4 ppm (–CONH–CH_2_–***CH_2_***–, C) correspond to the methylene chemical shifts in the repeat unit; the peaks at δ = 2.0 ppm (–CH_2_–***CH_2_***–CONH–, F) to the methylene chemical shift of α position in the amide group; the peaks at δ = 3.0 ppm (–OCNH–***CH_2_***–CH_2-_, D) to the methylene chemical shift adjacent to the amide group; the peak at δ = 2.1 ppm (–***CH_2_***–COOH, E) to the methylene chemical shift at the α position of the carboxyl group; and the peaks at δ = 7.7 ppm (–OC–N***H***–, H) and δ = 12.0 ppm (–COO***H***, I) to the chemical shifts of the amide hydrogen and terminal carboxylic acid group, respectively. All these shifts are consistent with the nylon-6 structure [16].

Figure 2b shows the map overlay of nylon-6 and nylon-6(*N*-methyl-30%), whose chemical shifts are basically similar to those of nylon-6 except that the shifts at δ = 2.8 ppm and δ = 2.9 ppm (–CH_2_–CON(***CH_3_***)–, *) represent the signals of the methyl group on the amide functional group at the terminal and in the main chain, respectively, which confirms that polymerisation between the *N*-methyl-caprolactam monomer and caprolactam had been successful. Figure 2c represents the map overlay of nylon-6(*N*-acetyl-30%) and nylon-6. The chemical shifts at δ = 1.6–1.7 ppm (–CO–N(CO***CH_3_***)–, *) represent the signals of the acetyl group confirming that *N*-acetyl-caprolactam monomer has been involved in the polymerisation reaction.

#### 3.1.2. FT-IR

**Figure 3:**
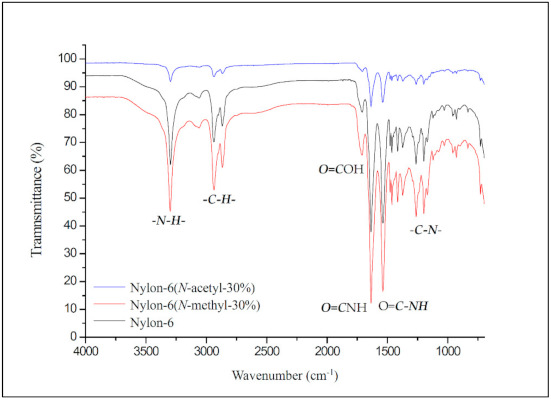
FT-IR spectra of nylon-6, nylon-6(*N*-methyl-30%), and nylon-6(*N*-acetyl-30%).

Figure 3 shows the FT-IR map overlay of nylon-6, nylon-6(*N*-methyl-30%), and nylon-6(*N*-acetyl-30%). The peak at 3295 cm^−1^ represents the absorption of the structural amide group (–CO–***NH***–), while the peaks at 2942 cm^−1^ and 2873 cm^−1^ represent the absorption of the structural methylene group in nylon-6. The absorption peaks at 1716 cm^−1^ and 1636 cm^−1^ can be attributed to the carbonyl stretching of adipic acid and the amide group of nylon-6, respectively. The peak at 1536 cm^−1^ corresponds to C–N stretching and that at 1263 cm^−1^ to amide bending [17]. No obvious absorption difference caused by *N*-substitution can be seen from the FT-IR spectra.

#### 3.1.3. ^13^C NMR

**Figure 4:**
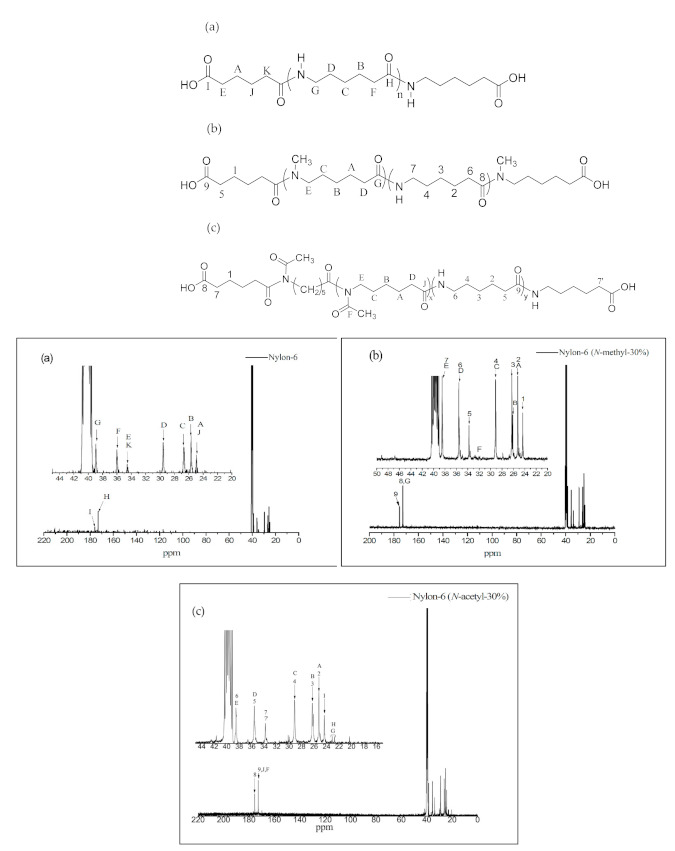
^13^C NMR spectra of (**a**) nylon-6, (**b**) nylon-6(*N*-methyl-30%), and (**c**) nylon-6(*N*-acetyl-30%). The letter and number represent chemical shift of each structural atom.

The ^13^C NMR spectra and structural assignments of nylon prepolymers are shown in Figure 4a–c. In Figure 4a, the chemical shifts of carbon in adipic acid and the methylene group can be seen at δ = 24.3 ppm [–CH_2_(***CH_2_***)_2_CH_2_–, A, J] and δ = 33.9 ppm [–***CH_2_***CO_2_H–***CH_2_***CONH–, E, K], respectively. At δ = 25.1 ppm [–(CH_2_)_3_–***CH_2_***–CH_2_–, B], δ = 26.1 ppm [– (CH_2_)_2_–***CH_2_***–(CH_2_)_2_–, C], δ = 28.9 ppm [–CH_2_–***CH_2_***–(CH_2_)_3_–, D], δ = 35.4 ppm [–***CH_2_***–CONH–, F], and δ = 38.3 ppm [–***CH_2_***–HNOC–, G], the chemical shifts of carbon in the methylene group of nylon-6 can be observed. The peak at δ = 171.9 ppm [–***CO***NH–, H] corresponds to the chemical shift of the carbonyl group in the amide group, while those at δ = 174.7 ppm [–***CO***OH, I] and 171.9 ppm [–***CO***NH, H] correspond to the chemical shifts of the carbonyl group of adipic acid and the amide group of nylon-6, respectively. The results are consistent with what is stated in the literature [18].

As Figure 4b shows, nylon-6(*N*-methyl-30%) copolymers all have the characteristic chemical shifts of nylon-6 block copolymers, such as those observed at δ = 25.1 ppm [– (CH_2_)_3_–***CH_2_***–CH_2_–, 2], δ = 26.1 ppm [–(CH_2_)_2_–***CH_2_***–(CH_2_)_2_–, 3], and δ = 28.9 ppm [–CH_2_–***CH_2_***–(CH_2_)_3_–, 4]. The chemical shift at δ = 32–33 ppm (–N–***CH***_3_, F), δ = 171.5–172.0 ppm (–***CO***NH–, –***CO***N(CH_3_)–; 8, G) and δ = 174.5 ppm (–***CO***OH, 9) can be attributed to the methyl substituent in nitrogen, the characteristic carbonyl chemical shift of polyamide, and the chemical signal of adipic acid, respectively.

The peaks at δ = 25.1 ppm [–***CH_2_***–CH_2_–CONH–, 2], δ = 26.2 ppm [–(CH_2_)_2_–***CH_2_***– (CH_2_)_2_–, 3], δ = 28.9 ppm [–CON–CH_2_–***CH_2_***–, 4], δ = 35.4 ppm [–***CH_2_***–CONH–, 5], and δ = 38.3 ppm [–CON–***CH_2_***–, 6] in Figure 4c represent the chemical shifts of carbon in the methylene group of nylon-6 and *N*-acetyl-caprolactam moieties. The peak at δ = 168.9–171.8 ppm represents the chemical shift of the amide carbonyl group in nylon copolymers, and that at δ = 174.5 ppm [HO***OC***–, 8] the characteristic chemical shift of the adipic acid end group.

### 3.2. Structural Analysis of PEBA

PEBA copolymers are prepared by melt condensation of acid-terminated nylon-6, nylon-6(*N*-methyl-X%), and nylon-6(*N*-acetyl-X%) at 230 °C using different molecular weights of PTMEG with titanium (IV) isopropoxide as the catalyst. The summarised results of the analysis are presented in Table 2. The FT-IR map overlay of PEBA 250, PEBA 250(*N*-Methyl-30%) and PEBA 250(*N*-Acetyl-30%) are shown in Supplementary Materials, Figure S4. In general, the absorptions are similar to those of nylon prepolymers except that a distinct ether stretching appears at 1101 cm^−1^ (*C*–***O–C***), which can be attributed to the incorporation of PTMEG.

#### 3.2.1. ^1^H NMR

**Figure 5:**
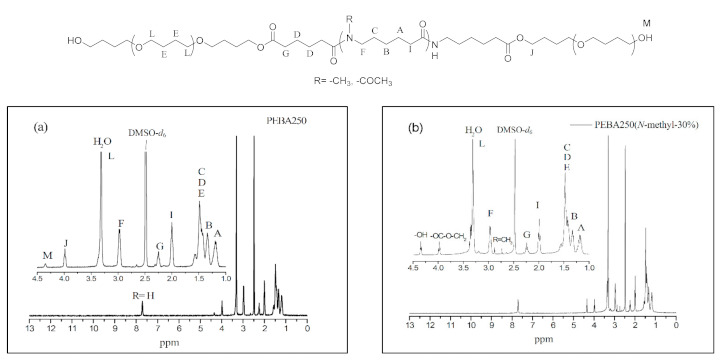

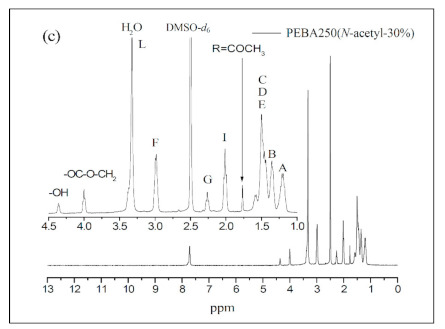
^1^H NMR spectra of (**a**) Polyether-*block*-amide (PEBA 250), (**b**) PEBA 250(*N*-methyl-30%), and (**c**) PEBA 250(*N*-acetyl-30%).

Figure 5 a–c show the ^1^H NMR spectra of PEBA 250, PEBA 250(*N*-methyl-30%), and PEBA 250(*N*-acetyl-30%). In Figure 5a, we can see that the chemical shifts of methylene groups at δ = 1.4–1.6 ppm (–C***H***_2_–CH_2_O–, E) and δ = 3.2–3.4 ppm (–CH_2_–C***H***_2_O–, L) are the signals from PTMEG. Moreover, the peak at δ = 4.0 ppm (–COO–***CH_2_***–, J) represents the chemical shift of a new ester group indicating that esterification had been successful. Likewise, the ^1^H NMR spectra in Figure 5b,c reveal that the two typical signals of *N*-methyl protons at δ = 2.7 ppm (–N–***CH***_3_) and *N*-acetyl protons at δ = 1.8 ppm (–N–CO–***CH***_3_) are also compatible with the structure. The relative content of polyether to polyamide were calculated based on the integrations of ester peak at 4.0 ppm and the methylene chemical shift adjacent to the amide group at 3.0 ppm. The results were summarized in Table 2. The ether content of PEBA-30% polymers (Entry 12, 15, 18, and 21) are generally lower than that of 10% and 20% analogues. It might be expected that the prepolymer with less steric hindrance could coordinate with catalysts more easily than the more steric one, thus accounting for the difference.

**Figure 6:**
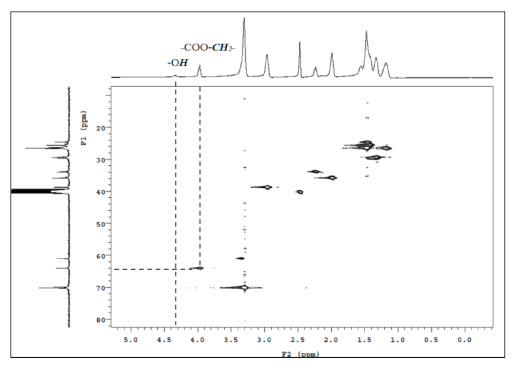
HMQC spectra of PEBA 250.

The chemical shift at δ = 4.4 ppm was found to be the characteristic peak of the terminal hydroxyl group (–O***H***, M), which is also present in the HMQC spectra of PEBA 250 (Figure 6). The point δ = 4.0 ppm (–COO–***CH_2_***–) in Figure 6 can correspond to δ = 63.5 ppm in ^13^C NMR whereas δ = 4.4 ppm (–O***H***) cannot correspond to any point in ^13^C NMR. Similar results can also be found in *N*-substituted derivatives indicating that esterification had occurred.

#### 3.2.2. XRD Analysis

In order to understand the impact of the substituent on the structural arrangement of the products, we performed XRD to analyse the crystallinity of PEBA and its *N*-substituted derivatives. The results are presented in Table 2.

**Figure 7:**
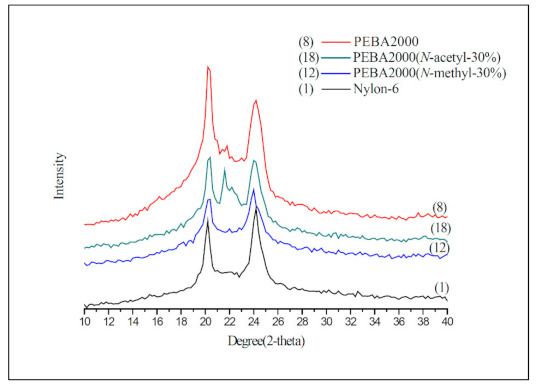
XRD spectra of Nylon-6, PEBA 2000, PEBA 2000(*N*-methyl-30%), and PEBA 2000(*N*-acetyl-30%).

Nylon-6 is a semi-crystalline polymer, whose crystalline phase can be divided into two phases: Phase α and Phase γ. The α crystalline phase (2θ: 20°, 23–24°) is mainly a fully extended or twisted nylon-6 structure and is arranged in an antiparallel fashion, while the γ crystalline phase (2θ: 21°) is the result of the amide group forming hydrogen bonds at a bond angle of 60° [19,20]. According to the crystallinity values presented in Table 2, the *N*-substituted group has broken the overall structural regularity of nylon-6, thereby decreasing its crystallinity with the substituent accounting for higher mole percentages (Entries 2–7). This phenomenon was also observed in the product structure of PEBA(*N*-methyl-X%) (Entries 10–15). Interestingly, in the case of PEBA(*N*-acetyl-X%), crystallinity first dropped and then increased. Comparing to the PEBA 2000, the crystallinity of PEBA 2000(*N*-acetyl-10%, -20%, and -30%) fell from 57.2% (Entry 8) to 31.3%, 34.9% (Entry 16,17) and then rebounded to 59.4% (Entry 18). It is presumed that as the proportion of *N*-acetyl-caprolactam increases, new hydrogen bonding between the carbonyl group of the *N*-acetyl moiety and the amide group can occur, which can result in a new arrangement of the γ form causing a rebound in the crystallinity. Figure 7 shows the XRD map overlay of nylon-6, PEBA 2000, PEBA 2000(*N*-methyl-30%), and PEBA 2000(*N*-acetyl-30%). As the figure shows, all samples exhibited diffractions at 2θ = 20.3 and 24.0, which correspond to the crystalline phase of the nylon-6 matrix; however, a small diffraction peak at 2θ = 21.5° was observed for PEBA 2000(*N*-acetyl-30%), which can be attributed to the γ crystalline form in the microstructures [21,22].

A similar phenomenon could also be observed in the PEBA 250 series; in PEBA 250 (Entry 9), PEBA 250(*N*-methyl-X%) (Entries 13–15), and PEBA 250(*N*-acetyl-X%) (Entries 19–21), the crystallinity of *N*-methyl fell from 74.3% (Entry 9) to 71.8%, 48.3% and 35.4% (Entries 13–15) as the substituent ratio was increased, whereas in *N*-acetyl cases, the crystallinity of PEBA 250(*N*-acetyl-10%, -20%, and -30%) fell from 74.3% (Entry 9) to 54.2%, 57.0% (Entries 19,20) and then rebounded to 79.7% (Entry 21).

### 3.3. Thermal Properties

#### 3.3.1. Thermal Properties of Nylon Prepolymers

The influence of the substituent on the crystallinity of the product can also manifest in the thermal properties of the product. Thus, DSC and TGA were performed to explore the effect of composition on the crystalline structure of PEBA copolymer. According to Table 2, as the ratio of the substituent monomer is increased, the melting point (*T*_m_) of nylon-6(*N*-methyl-X%) prepolymer decreases from 185 °C (Entry 1) to 178, 175 and 157 °C (Entries 2–4), whereas that of nylon-6(*N*-acetyl-X%) prepolymer (Entries 5–7) first decreases from 185 to 165 °C and then increases from 165 to 170 °C. This downward trend of the melting point is consistent with the change in crystallinity. With regard to thermal stability, the thermogravimetric analysis shows that *T*_d,5%_ falls as the proportion of the substituent monomer is increased. The principal degradation mechanism is generally believed to begin with the scission of the C(O)–NH bonds [23]. In general, the amide group (–CH_2_–***CON***–***CH_2_***–) in nylon is the least stable and is expected to break first. Because of the bond-dissociation energy of small molecules, the N–C bond-dissociation enthalpies (DH^o^_298_) of H_2_***N***–***C***(O)H stands at 413.7 kcal/mol, which is slightly higher than that of either *N*-methyl (CH_3_***N***–***C***(O)H) bond (403.7 kcal mol^−1^) or *N*-acetyl (H_2_***N***–***C***(O)CH_3_) bond (406.7 kcal mol^−1^ [24]. As a result, non-substituted nylon-6 prepolymer and PEBA co-polymer have high *T*_d,5%_ temperatures.

#### 3.3.2. Thermal Properties of PEBA

In the PEBA(*N*-methyl-X%) series (Entries 10–15), the melting points fell as the proportion of methyl-caprolactam monomer was increased. PEBA 250(*N*-methyl-30%) exhibited the most notable reduction in the melting point, which was as low as 157 °C. Thus, it can be considered for applications requiring low melting and foaming temperatures in scale up production, which justifies its use in the subsequent cross-linking reactions. In the series of PEBA(*N*-acetyl-X%) (Entries 16–21), the decomposition temperature (T_d,5%_) decreased with the incorporation of the acetyl-caprolactam group; however, this temperature showed an upward trend as the proportion of the acetyl-caprolactam content was increased. For example, unlike the T_d,5%_ of PEBA 2000 (Entry 8), the T_d,5%_ of PEBA 2000(*N*-acetyl-X%) first fell from 332 °C to 277 °C (Entry 16) and then rose to 285 °C (Entry 18). Likewise, in comparison with the T_d,5%_ of PEBA 250 (Entry 9), the T_d,5%_ of PEBA 250(*N*-acetyl-X%) first fell from 350 °C to 220 °C (Entry 19) and then increased to 253 °C (Entry 21). The trend of the decomposition temperature is similar to the trend of the crystallinity. In particular, although the regular arrangement of the original nylon-6 was disturbed by the introduction of the acetyl functional group as the proportions of the substituent monomers were increased, the acetyl functional group was responsible for the increase in the crystallinity of PEBA 2000(*N*-acetyl-30%) caused by intramolecular hydrogen bonding. Consequently, the thermal properties of PEBA 2000(*N*-acetyl-30%) also improved. It was also observed that the molecular weight of PTMEG affects the thermal properties of PEBA. The higher the molecular weight of PTMEG, the higher is the thermal stability of *N*-substituted nylon elastomers. With regard to T_d,5%_, the molecular weight of PTMEG had a higher influence on *N*-methyl functionalized PEBA than that of any *N*-acetyl functionalized analogue. When the 10% substituted group (Entries 10, 13, 16, and 19) is considered as an example, the T_d,5%_ of PEBA 250(*N*-methyl-10%) and PEBA 250(*N*-acetyl-10%) were 71 and 57 °C lower than the T_d,5%_ of PEBA 2000(*N*-methyl-10%) and PEBA 2000(*N*-acetyl-10%), respectively. As for the groups with 20% substitution (Entries 11 vs. 14, and 17 vs. 20), the difference increased to 120 and 29 °C and for the group with 30% substitution, the difference increased to 116 and 32 °C (Entries 13 vs. 16 and 19 vs. 22). Similar results were observed with regard to the melting points. With the group with the same percentage substitution, the molecular weight of PTMEG and the *T*_m_ of the nylon elastomers were lower.

### 3.4. Chemical Cross-Linking of PEBA

**Figure 8:**
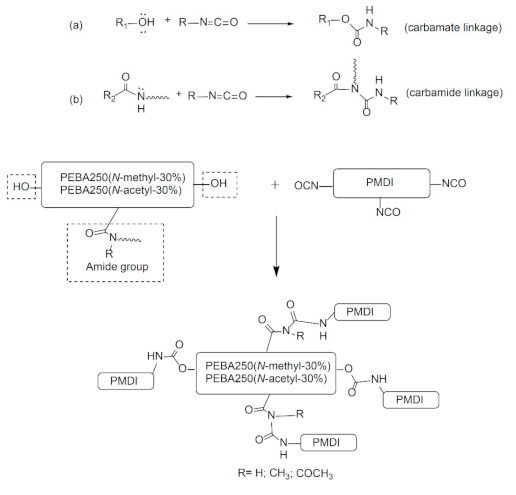
Cross-linking reaction of PEBA–poly(4,4′-methylenebis(phenyl isocyanate) (PMDI) (a) through carbamate linkage (b) through carbamide linkage.

A good cross-linking reaction can improve the mechanical strength of the elastic foaming materials and enable the melt to stay stable during foaming without letting the cells burst. To meet the requirements pertaining to low-temperature foaming, we chose elastomers made of PEBA 250(*N*-methyl-30%) and PEBA 250(*N*-acetyl-30%) which have a low melting point for chemical cross-linking with poly(4,4′-methylenebis(phenyl isocyanate) (PMDI). Figure 8 shows the schematic diagram of the cross-linking reaction formed through carbamate or carbamide linkage.

**Figure 9:**
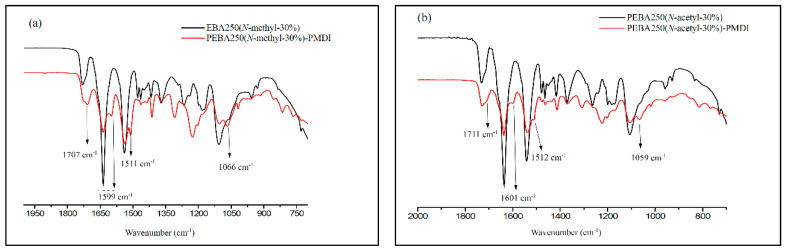
FT-IR spectra of (**a**) PEBA 250(*N*-methyl-30%), PEBA 250(*N*-methyl-30%)–PMDI and (**b**) PEBA 250(*N*-acetyl-30%), PEBA(*N*-acetyl-30%)–PMDI.

The cross-linking reaction was carried out in an argon-filled environment under molten conditions. The temperature was slowly increased from 160 to 165 °C to melt the PEBA, following which 30–37 wt% of PMDI (NCO content 23%) was added and stirred evenly for 15 min. Heating was then stopped, while maintaining the argon-filled environment to enable cooling. The product was purified by thorough wash with acetone and isolated as brownish yellow solid in quantitative yield after drying by vacuum for 2 h. The swelling ratio of the cross-linked polymer was estimated 150% after stirring the samples with DMSO for 15 h at 25 °C. Figure 9 shows the FT-IR spectra of PEBA 250(*N*-methyl-30%)-PMDI and PEBA 250(*N*-acetyl-30%)–PMDI. As Figure 9a shows, the resulting product PEBA 250(*N*-methyl-30%)–PMDI has a new absorption peak at 1707 cm^−1^, which can be attributed to the formation of carbamate (–O–***CO***–NH–, stretch), while the new peaks at 1599 cm^−1^ and 1511 cm^−1^ correspond to the carbamide C–N stretching and C–H bending, respectively [25,26]. The new absorption peak at 1066 cm^−1^ can be assigned to ether linkages (–C–***O–C***–, stretch) in PMDI after the cross-linking reaction has occurred. Similarly, in Figure 9b, the new peaks at 1711, 1601, 1512, and 1059 cm^−1^ also confirm that cross-linking reactions had occurred. After the cross-linking reaction, the T_d,5%_ of PEBA 250(*N*-methyl-30%)–PMDI significantly increased by 78 °C, i.e., from 199 to 277 °C, while the T_d,5%_ of PEBA 250(*N*-acetyl-30%)–PMDI increased only by 6 °C, i.e., from 253 to 259 °C (Table 2, Entries 15 vs. 22, 21 vs. 23). No distinct glass transition behaviour is observed, and the crystalline detraction peaks were no longer present, either (Figure S5.). It is thought that after crosslinking reaction, the microstructures might have restricted the segmental movement of PEBA 250(*N*-Methyl-30%)–PMDI and therefore exhibit no obvious phase transition.

The acetyl-substituted group has electro-withdrawing characteristics, which decrease the nucleophilicity of amide nitrogen, thereby making it more difficult for the cross-linking reactions to take place, while the methyl-substituted group has electro-donating characteristics, which increase the nucleophilicity of amide nitrogen, enabling cross-linking reactions to take place successfully. The DSC results also show that PEBA 250(*N*-methyl-30%)–PMDI has no distinct melting point, which indicates that the crystal structure has broken during the cross-linking reactions, whereas PEBA 250(*N*-acetyl-30%)–PMDI continues to exhibit the melting point at 149 °C, confirming that its cross-linking reaction is incomplete.

## 4. Conclusions

In this study, we have successfully synthesized a series of new *N*-substituted nylon copolymers with polyamide-6 as the hard segment and polyether as the soft segment. Studies have shown that the modification of the amide functional group can break the regularity of the overall structural arrangement of the original nylon-6 and that its crystallinity gets reduced as the proportion of the substituent is increased, which tallies with DSC data. In the PEBA(*N*-acetyl-X%) series, because the *N*-acetyl functional group can also form intramolecular hydrogen bonds as the proportion of the substituent monomers is increased, the crystallinity of the product first decreases and then increases. The rebound in crystallinity can be attributed to the new intramolecular hydrogen bonding formed, which can produce a new arrangement of the γ crystalline form in the polymer, as shown by the XRD. A similar trend can also be observed in their pyrolysis temperatures. After the cross-linking reaction with PMDI, the T_d,5%_ of PEBA 250(*N*-methyl-30%) increased by 78 °C, whereas that of PEBA 250(*N*-acetyl-30%) did not show such a notable increase indicating that the electro-donating group can increase the nucleophilicity of nitrogen, which will be beneficial to cross-linking reactions. In contrast, the electro-withdrawing group can reduce the nucleophilicity of nitrogen and thereby adversely affect the cross-linking reaction.

## Figures and Tables

**Table 1:** Feed ratio and molecular weight data of the nylon prepolymer.

Entry	Compound	Caprolactam	*N-*Methyl-ε- Caprolactam	*N-*Acetyl-ε- Caprolactam	M_*n*, (NMR)_	Yield (%)
		Feed (mol)				
1	Nylon-6	0.075	-	-	883	66
2	Nylon-6(*N-*methyl-10%)	0.0675	0.0075	-	986	45
3	Nylon-6(*N-*methyl-20%)	0.06	0.015	-	938	51
4	Nylon-6(*N-*methyl-30%)	0.0525	0.0225	-	1061	48
5	Nylon-6(*N-*acetyl-10%)	0.0675	-	0.0075	974	46
6	Nylon-6(*N-*acetyl-20%)	0.06	-	0.015	1019	50
7	Nylon-6(*N-*acetyl-30%)	0.0525	-	0.0225	962	45

**Table 2:** Thermal properties of Nylon, PEBA, PEBA(*N*-methyl-X%), and PEBA(*N*-acetyl-X%).

Entry	Compound	T_d,5%_	T_d,10%_	T_d,max%_	T_m_	Char	*X*_c_^1^	Ether ^2^
		(°C)	(°C)	(°C)	(°C)	%	%	wt%
1	nylon-6	310	324	396	185	2.33	65.6	-
2	nylon-6(*N*-methyl-10%)	299	318	390	178	3.68	65.9	-
3	nylon-6(*N*-methyl-20%)	293	309	428	175	2.37	59.1	-
4	nylon-6(*N*-methyl-30%)	285	304	433	157	4.47	39.9	-
5	nylon-6(*N*-acetyl-10%)	269	294	342	165	2.08	65.9	-
6	nylon-6(*N*-acetyl-20%)	258	283	444	172	2.82	63.2	-
7	nylon-6(*N*-acetyl-30%)	253	283	451	170	6.85	51.7	-
8	PEBA 2000	332	354	423	189	3.14	57.2	87.6
9	PEBA 250	350	376	417	179	3.92	74.3	40.5
10	PEBA 2000(*N*-methyl-10%)	300	315	388	193	3.9	60.6	84.4
11	PEBA 2000(*N*-methyl-20%)	323	347	390	182	6.95	57.2	85.9
12	PEBA 2000(*N*-methyl-30%)	315	335	395	182	7.49	35.5	48.5
13	PEBA 250(*N*-methyl-10%)	229	274	350	175	19.08	71.8	41.4
14	PEBA 250(*N*-methyl-20%)	203	244	321	170	24.48	48.3	44.3
15	PEBA 250(*N*-methyl-30%)	199	234	355	157	16.28	35.4	28.7
16	PEBA 2000(*N*-acetyl-10%)	277	287	407	179	4.54	31.3	84.5
17	PEBA 2000(*N*-acetyl-20%)	276	288	403	179	7.15	34.9	70.7
18	PEBA 2000(*N*-acetyl-30%)	285	298	405	179	6.53	59.4	57.1
19	PEBA 250(*N*-acetyl-10%)	220	256	371	159	12.65	54.2	36.1
20	PEBA 250(*N*-acetyl-20%)	247	290	370	166	13.88	57	44.3
21	PEBA 250(*N*-acetyl-30%)	253	292	364	164	13.78	79.7	33.2
22	PEBA 250(*N*-methyl-30%)-PMDI	277	295	419	-	5.41	-	-
23	PEBA 250(*N*-acetyl-30%)-PMDI	259	281	349	149	11.99	-	-

1. XRD crystallinity. 2. Polyether weight content.

## Data Availability

The data presented in this study are available from the corresponding author on reasonable request.

## Supplementary Materials

The following are available online at https://www.mdpi.com/1996-1944/14/4/773/s1. Figure S1: Experimental Set-up of Nylon Prepolymer. Figure S2: Experimental Set-up of Polyether-*block*-amides (PEBA). Figure S3: Photographs of products: (1) nylon-6, (2) nylon-6(*N*-acetyl-10%), (3) nylon-6(*N*-methyl-10%), (4) PEBA 2000, (5) PEBA 2000(*N*-acetyl-10%), (6) PEBA 250(*N*-methyl-30%), (7) PEBA 250, (8) PEBA 250(*N*-acetyl-30%), and (9) PEBA 250(*N*-methyl-10%). Figure S4: FT-IR spectra of PEBA 250, PEBA 250(*N*-Methyl-30%), and PEBA 250(*N*-Acetyl-30%). Figure S5: XRD spectra of PEBA 250(*N*-methyl-30%)–PMDI.

## References

[1] Sarwar Z., Krugly E., Danilovas P.P., Ciuzas D., Kauneliene V., Martuzevicius D. (2019). Fabrication and characterization of PEBA fibers by melt and solution electrospinning. J. Mater. Res. Technol..

[2] Sheth J.P., Xu J., Wilkes G.L. (2003). Solid state structure-property behavior of semicrystalline poly(ether-block-amide) PEBAX thermoplastic elastomers. Polymer.

[3] Flesher J.R., Seymour R.B., Kirshenbaum G.S. (1986). Pebax^®^ polyether block amide—A new family of engineering thermoplastic elastomers. High Performance Polymers: Their Origin and Development.

[4] Mandal M.K., Bhattacharya P.K. (2006). Poly(ether-block-amide) membrane for pervaporative separation of pyridine present in low concentration in aqueous solution. J. Memb. Sci..

[5] Sridhar S., Kalyani S., Ravikumar Y.V.L., Muralikrishna T.S.V.N. (2010). Performance of composite membranes of poly(ether-block-amide) for dehydration of rthylene glycol and ethanol. Sep. Sci. Technol..

[6] Sridhar S., Suryamurali R., Smitha B., Aminabhavi T.M. (2007). Development of crosslinked poly(ether-block-amide) membrane for CO_2_/CH_4_ separation. Colloids and Surf. A Physicochem. Eng. Aspects.

[7] Tena A., Shishatskiy S., Filiz V. (2015). Poly(ether-amide) vs. poly(ether-imide) copolymers for post-combustion membrane separation processes. RSC Adv..

[8] Zdrahala R., Firer E.M., Fellers J.F. (1977). Block copolymers of poly(m-phenylene isophthalamide) and poly(ethylene oxide) or polydimethylsiloxane: Synthesisand general characteristics. J. Polym. Sci. A. Polym. Chem..

[9] Gaymans R.J., Schwering P., de Haan J.L. (1989). Nylon 46-polytetramethylene oxide segmented block copolymers. Polymer.

[10] Castaldo L., Maglio G., Palumbo R. (1978). Synthesis of polyamide-polyether block copolymers. J. Polym. Sci. Polymer Lett. Ed..

[11] Kimura Y., Sugihara N., Taniguchi I. (1983). Novel polycondensations via poly(oxyethy1ene) diglycolic acid diamine salts. Macromolecules.

[12] Rasmussen J.K., Smith II H.K. (1983). Polyamide–polyether copolymers: A new family of impact-resistant thermoplastics. J. Appl. Polym. Sc..

[13] Xu S., Ye L. (2015). Preparation and properties of monomer casting Nylon-6/PEO blend prepared via in situ polymerization. Polym. Eng. Sci..

[14] Rwei S.P., Ranganathan P., Chiang W.Y., Lee Y.H. (2018). Synthesis of low melting temperature aliphatic-aromatic copolyamides derived from novel bio-based semi aromatic monomer. Polymers.

[15] Peyravi M., Babaluo A.A., AkhfashArdestani M., RazaviAghjeh M.K., Pishghadam S.R., Hadi P. (2010). Study on the synthesis of poly(ether-block-amide) copolymer based on nylon6 and poly(ethylene oxide) with various block lengths. J. Appl. Polym. Sci..

[16] Wang C., Hu F., Yang K., Hu T., Wang W., Deng R., Jiang Q., Zhang H. (2015). Synthesis and properties of star-branched Nylon6 with hexafunctionalcyclotriphosphazene core. RSC Adv..

[17] Wang G., Xue B. (2010). Synthesis and characterization of poly(ether-block-amide) and application as permanent antistatic agent. J. Appl. Polym. Sci..

[18] Davis R.D., Jarrett W.L., Mathias L.J. (2001). Solution 13C NMR spectroscopy of polyamide homopolymers (Nylons 6, 11, 12, 66, 69, 610 and 612) and several commercial copolymers. Polymer.

[19] Dasgupta S., Hammond W.B., Goddard W.A. (1996). Crystal structures and properties of nylon polymers from theory. J. Am. Chem. Soc..

[20] Xu S., Ye L. (2015). Synthesis and properties of monomer cast Nylon-6-b-polyether amine copolymers with different structures. RSC Adv..

[21] Wang C., Tsou S.Y., Lin H.S. (2012). Brill transition of nylon-6 in electrospun nanofibers. Colloid Polym. Sci..

[22] Fornes T.D., Paul D.R. (2003). Crystallization behavior of nylon 6 nanocomposites. Polymer.

[23] Lehrle R.S., Parsons I.W., Rollinson M. (2000). Thermal degradation mechanisms of Nylon 6 deduced from kinetic studies by pyrolysis-g.c. Polym. Degrad. Stabil..

[24] Marochkin I.I., Dorofeeva O.V. (2012). Amide bond dissociation enthalpies: Effect of substitution on N–C bond strength. Comput. Theor. Chem..

[25] Chung Y.C., Choi J.W., Chung H.M., Chun B.C. (2012). The MDI-mediated lateral crosslinking of polyurethane copolymer and the impact on tensile properties and shape memory effect. B. Korean Chem. Soc..

[26] Wong C.S., Badri K.H. (2012). Chemical analyses of palm kernel oil-based polyurethane prepolymer. Mater Sci Appl..

