# Supplementary material for: Synthesis and Characterization of N-Substituted Polyether-Block-Amide Copolymers
Source: Materials (Basel). 2021 Feb 6;14(4):773. doi: 10.3390/ma14040773 (PMC7915099; doi:10.3390/ma14040773)
Supplement: Supplementary file 1 [file materials-14-00773-s001.pdf]

materials

MDPI

Supplementary

Synthesis and Characterization of *N*-Substituted Polyether-*Block*-Amide Copolymers

Jyun-Yan Ye [1], Kuo-Fu Peng [2], Yu-Ning Zhang [1], Szu-Yuan Huang [2] and Mong Liang [1,*]

1 Department of Applied Chemistry, National Chia-Yi University, Chia-Yi 600, Taiwan; s1000268@gm.pu.edu.tw (J.-Y.Y.); s1080246@mail.ncyu.edu.tw (Y.-N.Z.)
2 Department of Footwear Technology, Footwear & Recreation Technology Research Institute, Taichung 407, Taiwan; 0828@bestmotion.com (K.-F.P.); 0150@bestmotion.com (S.-Y.H.)
* Correspondence: mliang@mail.ncyu.edu.tw; Tel.: +886-52717952

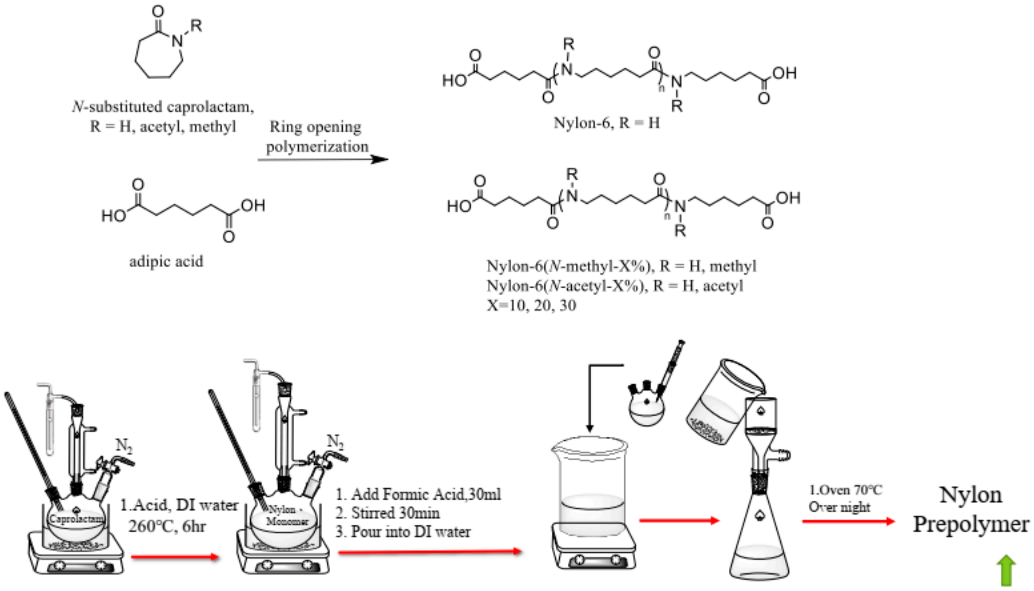

Figure S1. Experimental Set-up of Nylon Prepolymer.

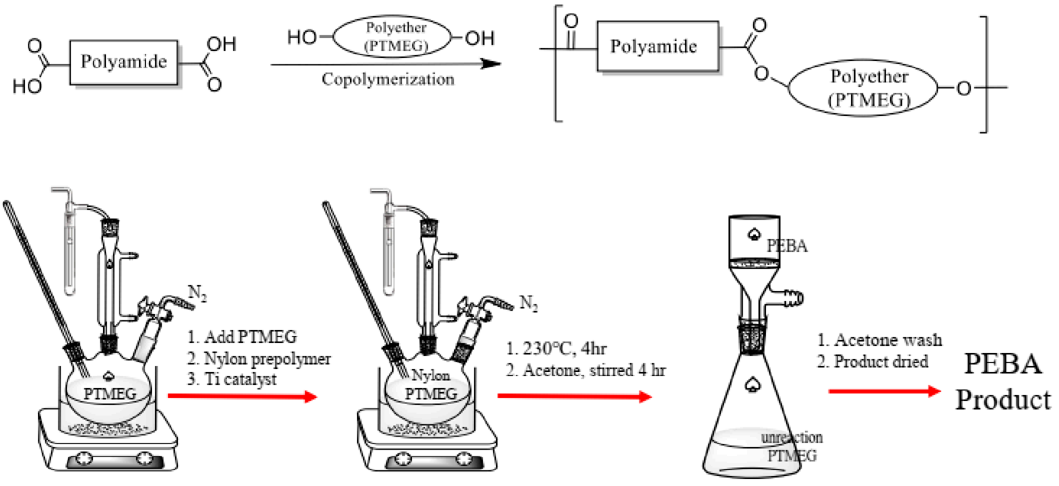

Figure S2. Experimental Set-up of Polyether-*block*-amides (PEBA).

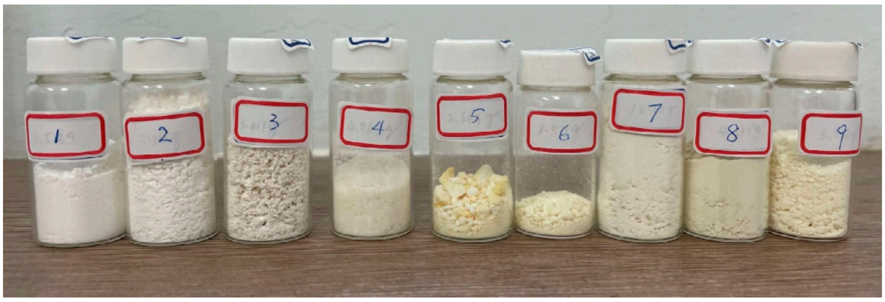

Figure S3. Photographs of products: (**1**) nylon-6, (**2**) nylon-6(*N*-acetyl-10%), (**3**) nylon-6 (*N*-methyl-10%), (**4**) PEBA 2000, (**5**) PEBA 2000(*N*-acetyl-10%), (**6**) PEBA 250(*N*-methyl-30%), (**7**) PEBA 250, (**8**) PEBA 250(*N*-acetyl-30%), (**9**) PEBA 250(*N*-methyl-10%).

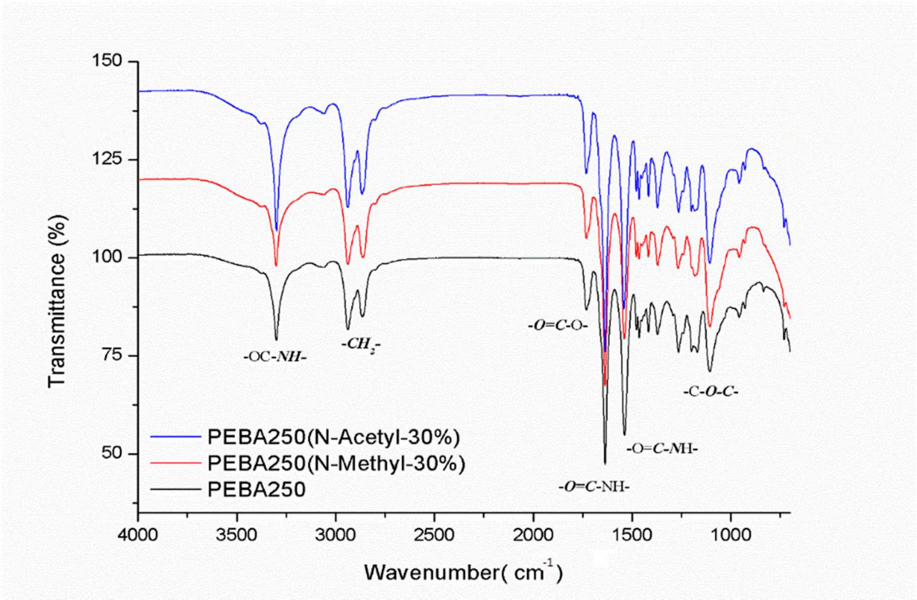

Figure S4. FT-IR spectra of PEBA 250, PEBA 250(*N*-methyl-30%), PEBA 250(*N*-acetyl-30%).

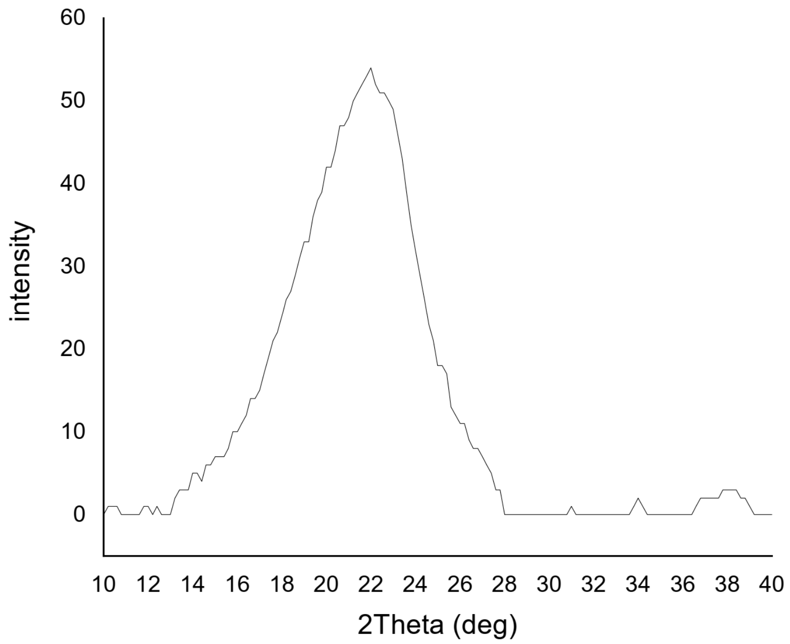

Figure S5. XRD spectra of PEBA 250(*N*-methyl-30%)–PMDI.
